# ‘I feel trapped in my safe clothes’: The impact of tactile hyper-sensitivity on autistic adults

**DOI:** 10.1177/13623613251366882

**Published:** 2025-10-07

**Authors:** Amanda Ferrer Knight, Deirdre Birtles

**Affiliations:** 1 Royal Holloway, University of London, UK

**Keywords:** appearance, autism, clothing, self-esteem, sensory processing

## Abstract

Clothing-related tactile hyper-sensitivity has been frequently reported among autistic people, but research investigating how this affects autistic adults is limited. This mixed-methods study investigated the impact of clothing-related tactile hyper-sensitivity on autistic adults’ lives, focusing on appearance dissatisfaction, appearance awareness, self-esteem and self-expression through clothing. An online survey was completed by 86 autistic adults in the United Kingdom. Regression analysis revealed a significant predictive relationship between tactile hyper-sensitivity and appearance dissatisfaction. Moderation analysis revealed a significant negative relationship between tactile hyper-sensitivity and self-esteem, moderated by appearance dissatisfaction. Moderation of the relationship between appearance dissatisfaction and self-esteem by appearance awareness was not significant, but both appearance constructs were independent negative predictors of self-esteem. Three themes relating to the impacts of clothing-related tactile sensitivity were generated through thematic analysis of open-ended questions: ‘Negative consequences’, ‘Managing clothing-related tactile sensitivity’ and ‘The emotional value of clothing’. Altogether, the findings indicate that clothing-related tactile sensitivity is prevalent and can impact autistic adults’ appearance satisfaction, self-esteem, clothing options and choices, and self-expression through clothing. This highlights the need for a wider choice and availability of sensory-friendly clothing and further research exploring the diverse effects of tactile hyper-sensitivity on autistic people’s lives, including mental health outcomes and employment opportunities.

**Lay abstract:**

Many autistic people experience strong reactions to sensory information, such as certain sounds or smells. Tactile hyper-sensitivity is the strong, negative reaction to touch. Previous research suggests autistic children experience tactile hyper-sensitivity towards clothing, such as disliking labels or seams touching them. However, little is known about tactile hyper-sensitivity towards clothing in autistic adults and how this affects how they see themselves, feel about their appearance and express themselves through clothing. This study explored this by asking 86 autistic adults in the United Kingdom to complete an online survey. The results showed that autistic adults with a higher level of tactile hypersensitivity are more likely to be dissatisfied with their appearance, and that higher tactile hyper-sensitivity is also linked to lower self-esteem among autistic adults who are unhappy with how they look. Autistic adults with greater awareness and dissatisfaction with their appearance were also found to have lower self-esteem. In addition, autistic adults shared with us that sensory sensitivity towards clothing can negatively affect how they feel physically and emotionally, and their ability to take part in activities such as formal social events. Autistic adults have different ways of managing this, such as buying multiple of the same sensory-friendly clothing item, inspecting clothes in shops before buying them and only wearing clothes that feel comfortable. Being able to wear clothes they like and that reflect who they are is important for many autistic adults; but difficulties finding comfortable clothing left some autistic adults feeling frustrated, unhappy with their appearance and less confident. Together, the findings suggest that access to sensory-friendly clothing is very important for autistic adults’ well-being. These findings are relevant for mental health professionals working with autistic adults, clothing brands and workplaces with a dress code or uniform.

Approximately 1% of adults in the United Kingdom are autistic (Brugha et al., 2011). Differences in sensory processing are commonly reported among autistic people, with estimates suggesting a prevalence as high as 94% (Crane et al., 2009; MacLennan, O’Brien, et al., 2022). This includes experiencing hypo-sensitivity (i.e. unresponsiveness to sensory stimuli), hyper-sensitivity (i.e. avoidance or strong negative reactions to sensory stimuli) and having a strong interest in specific sensory input (e.g. a visual fascination with lights) more frequently than neurotypical (NT) individuals (American Psychiatric Association, 2013; Balasco et al., 2020; Ben-Sasson et al., 2019). Autistic people also experience hyper-sensitivity more often than individuals with other developmental or clinical conditions (Ben-Sasson et al., 2019). Although the exact mechanisms remain unclear, these sensory processing differences are most likely due to variations in sensory-dedicated neural circuits in the brain, including anatomical differences in primary sensory regions and amygdala (Green et al., 2015; Robertson & Baron-Cohen, 2017).

Experiencing involuntary overstimulation of the senses or ‘sensory overload’ due to hyper-sensitivity can lead to serious emotional distress, cognitive difficulties and physical discomfort or pain. For example, autistic adults have reported feeling nauseous after eating certain food textures, physical pain after being hugged, difficulties concentrating due to noise, and experiencing shutdowns in intense sensory environments (Charlton et al., 2021; MacLennan, O’Brien, et al., 2022; Robertson & Simmons, 2015). Accordingly, research has found hyper-sensitivity to be correlated with anxiety in autistic adults (Hwang et al., 2020; Syu et al., 2020; Verhulst et al., 2022), as well as mental health difficulties, somatisation and sleep difficulties in autistic children (Green & Ben-Sasson, 2010; MacLennan et al., 2021; Mazurek & Petroski, 2014; Rossow et al., 2021).

Hyper-sensitivity can be a barrier to optimal engagement in many areas of life. Tactile hyper-sensitivity has been described as one of the reasons why autistic adults may have difficulties going to crowded spaces such as supermarkets where there may be unexpected physical contact (MacLennan, Woolley, et al., 2022); maintaining personal hygiene such as oral care due to the physical sensations (Mirsky et al., 2021); and accessing healthcare and physical examinations which involve being touched (Doherty et al., 2022; Mirsky et al., 2021). Autistic individuals have also reported struggling with tactile hyper-sensitivity towards clothing, in particular having a strong reaction to disliked fabrics touching their body (Kyriacou et al., 2021; MacLennan, O’Brien, et al., 2022; Robledo et al., 2012). Wearing clothes deemed to be uncomfortable can cause significant physical discomfort and emotional distress such as itchy skin, anxiety and irritability (Kyriacou et al., 2021). Consequently, lack of suitable apparel can be a barrier to social participation and everyday life activities, particularly in settings with a required dress code such as school, sports and special occasions (Edwards et al., 2024; Jordaan et al., 2023; Kyriacou et al., 2021). Qualitative studies suggest that autistic adults who experience hyper-sensitivity prefer softer, more flexible clothes and fabrics such as satin or cotton; and dislike tags, seams and abrasive fabrics such as hessian or wool (Kyriacou et al., 2021; MacLennan, O’Brien, et al., 2022). In online Autistic-led spaces, autistic adults have self-reported a sensory aversion to socks, synthetic fabrics, clothing tags and tight clothing such as jeans (redscorpio98, 2021; Pandora_Chaos353, 2021).

Clothing and fashion are conceptualised in society as a way of visually expressing one’s identity, including a person’s culture, religion, personality, gender identity, mood and interests (Ruggerone, 2017). Wearing clothing that expresses individuality is linked to increased self-esteem and higher satisfaction with one’s clothing among women in the general population (Tiggemann & Lacey, 2009). Throughout history, clothing has been an important identity-signalling tool and form of self-expression for members of oppressed groups, such as the LGBTQ+ community (Hester & Hehman, 2023). People with mental and physical disabilities have also described clothing as important for expressing their identity, building confidence and feeling attractive (Esmail et al., 2020; McBee-Black & Ha-Brookshire, 2018). Hyper-sensitivity towards clothing means that sometimes autistic people have to choose between their desired appearance and clothing style, and being comfortable and able to engage with everyday life (Kyriacou et al., 2021). This suggests that clothing-related hyper-sensitivity may limit the use of clothing as a form of self-expression and lead to autistic adults becoming less satisfied with their appearance.

Self-esteem can be defined as a person’s evaluation of their worth or value as a person (Van der Cruijsen & Boyer, 2020). Autistic children and adults tend to report lower self-esteem than non-autistic people (Nguyen et al., 2020; Van der Cruijsen & Boyer, 2020). The pertinent literature suggests that masking can contribute to lower self-esteem (Evans et al., 2023; Hull et al., 2017), whereas social support and positive perceptions of autism and autistic people are associated with higher self-esteem (Cooper et al., 2017; Nguyen et al., 2020). Autistic adults’ feeling of self-worth may also be adversely affected by the emotional and physical discomfort, and barriers to participation and self-expression caused by clothing-related hyper-sensitivity. Clothing-related hyper-sensitivity may particularly affect the self-esteem of autistic adults who are unhappy with their appearance. Due to low self-esteem being associated with mental health difficulties such as depression, anxiety and suicidality (Arwert & Sizoo, 2020; Cooper et al., 2017; Sowislo & Orth, 2013), which are disproportionately common among autistic people (Cassidy et al., 2020; Lai et al., 2019), understanding the potential role of various factors such as sensory processing differences on self-esteem is crucial.

It is important to mention that autistic individuals can face significant pressure regarding their presentation to the outside world. Autistic people are often forced to mask and suppress their autistic traits due to stigma, safety and discrimination concerns, and to obtain and participate in opportunities which may be inaccessible if they are ‘visibly autistic’ (Hull et al., 2017). This can result in mental, physical and emotional exhaustion due to the required concentration and self-control, and can impact autistic individuals’ self-perception, identity and sense of authenticity (Chapman et al., 2022; Evans et al., 2023; Hull et al., 2017). Bearing in mind that clothing influences how a person is perceived by others (Hester & Hehman, 2023), autistic adults may feel considerable pressure to meet socio-cultural standards of appearance in order to ‘fit in’ and prevent discrimination. For example, autistic women have described facing pressure to conform to gender roles and stereotypical presentations of femininity in terms of clothing and makeup (Kanfiszer et al., 2017; Seers & Hogg, 2021), and that dressing differently to non-autistic peers could result in social exclusion and disapproval (Mo et al., 2022). In a study by Healy et al. (2021), physical appearance was described as ‘a means to fit in’ with NTs by autistic adults, as they considered that being attractive and thin led to greater acceptance by others. Autistic adults who feel pressure to conform to socio-cultural standards of appearance may have heightened awareness of their appearance. In turn, heightened appearance awareness may be associated with lower self-esteem if dissatisfied with one’s appearance. To date, no research has investigated appearance awareness among autistic people; however, in the general population, increased appearance awareness moderates the relationship between appearance dissatisfaction and self-consciousness towards appearance (Moss & Rosser, 2012).

A better understanding of sensory processing has been highlighted as a top 10 research priority by the autistic community and is important for improving autistic people’s well-being and quality of life (Autistica, 2016; Cage et al., 2024). This study aims to quantitatively investigate the relationship between clothing-related tactile hyper-sensitivity, appearance dissatisfaction, appearance awareness and self-esteem, as well as qualitatively explore the impact of clothing-related tactile sensitivity on autistic adults’ lives and self-expression through clothing. A mixed-methods design was chosen in order to reduce the limitations of quantitative-only research and to collect in-depth findings which reflect participants’ real-life experiences.

Based on reports that clothing-related tactile sensitivity leads some autistic adults to choose comfort over their desired appearance (Kyriacou et al., 2021) and the idea that appearance dissatisfaction may increase vulnerability to negative self-perception when adversely impacted by clothing-related hyper-sensitivity, it is hypothesised that:

1. Higher levels of tactile hyper-sensitivity will be associated with higher appearance dissatisfaction.
2. There will be a significant predictive relationship between tactile hyper-sensitivity and self-esteem, moderated by appearance dissatisfaction. Higher tactile hyper-sensitivity will be associated with lower self-esteem in autistic adults who are dissatisfied with their appearance.

Furthermore, based on the research of Moss and Rosser (2012), it is hypothesised that:

3. There will be a significant predictive relationship between appearance dissatisfaction and self-esteem, moderated by appearance awareness. Higher appearance dissatisfaction will be associated with lower self-esteem in autistic adults who are highly aware of their appearance.

## Methods

### Participants

Eighty-six participants were recruited through opportunity sampling using social media; a university participant pool including students, staff and general public; and organisations supporting autistic people which shared the study via their social media, newsletter or website. Participants were eligible to take part if they were 18 years old or older, lived in the United Kingdom and formally identified as Autistic. Participants who did not meet the criteria and those who reported having an eating disorder (*n* = 1) were excluded from the analysis. Negative self-evaluation and body image disturbance are key features of eating disorders (American Psychiatric Association, 2013) and inclusion of participants with these conditions could skew the appearance-related results. There was no previous research available to guide the sample size calculation. Sample size was based on an a priori estimate to detect a medium effect size (*f*^2^ = 0.15; Cohen, 1992) with α = 0.05 and 0.8 power for a regression with a single predictor, which indicated 55 participants were required (G*Power 3.1.9.7; Faul et al., 2007). The final sample of 86 participants was sufficient to detect a medium effect for the simple regression and moderation analyses. Due to time restrictions on the recruitment window, it was not possible to obtain a larger sample sufficient for detecting a small effect size (*f*^2^ = 0.02, *N* = 395; Cohen, 1992).

During recruitment, there were two instances of a sudden, large influx of responses submitted simultaneously or within a few minutes, suggesting spamming of the survey. To ensure data was trustworthy, responses which met at least two of the following criteria were removed prior to data analysis: an IP address located outside of the United Kingdom; an IP address which completed the study multiple times; completing the study in under 7 min; responding inconsistently to multiple items (e.g. strongly agreeing that they are satisfied with their appearance, then strongly agreeing that they dislike the way the look); submitting identical complex sentences to the open-ended questions as another participant; or reporting a significant language barrier. This led to data from 57 different IP addresses being deleted.

The final sample included 49 female, 25 male and 11 non-binary participants. One participant did not report their gender. The mean age was 32.12 (SD = 12.41) years. Participants reported their ethnicity as: White (*n* = 76, 88.4%), Asian (*n* = 2, 2.3%), mixed ethnic background (*n* = 6, 7.0%) and other ethnic groups (*n* = 2, 2.3%). Regarding employment, 32 participants (37.2%) were part-time or full-time students, 37 (43.0%) were in part-time or full-time employment, six (7.0%) were unemployed, one was retired (1.2%) and 10 (11.6%) were unable to work. Data on socioeconomic status and educational attainment level was not collected.

Participants’ scores on the three ‘social bifactor’ subscales of the Comprehensive Autism Trait Inventory (CATI; English et al., 2021) were above the recommended cut-offs: Social Interaction (Median = 28, Range = 10–35), Communication (Median = 23, Range = 12–32) and Camouflage (Median = 27, Range = 9–35). These subscales were used for characterising the sample rather than control variables in analyses as the current study focuses on a non-social autistic trait dimension (tactile sensitivity) and previous research (English et al., 2021) suggests relatively weak correlations between the social bifactor subscales and the CATI Sensory Sensitivity subscale. Thirty-eight participants had additional mental or neurodevelopmental conditions. The most frequent were anxiety disorders (*n* = 14), attention-deficit hyperactivity disorder (*n* = 14) and depression (*n* = 13). Other co-occurring conditions included Tourette’s syndrome, obsessive-compulsive disorder, emotionally unstable personality disorder, post-traumatic stress disorder, sensory processing disorder, dyspraxia, developmental language disorder, specific learning difficulties and conduct disorder.

### Ethics

This project was granted ethical approval by the Department of Psychology at Royal Holloway, University of London. All participants read a participant information sheet and provided written informed consent prior to participating. In exchange for taking part in the study, participants could enter a draw to win one of two £25 Amazon vouchers. Participants’ contact details were kept separate from the data and were deleted after the draw.

### Materials

#### Quantitative

##### Importance of fashion style

Participants completed a closed question written by the first author (A.F.K.) to quantitatively measure the importance of fashion style to participants and explore relationships between this and variables measuring appearance self-schema. The closed question was ‘How important is having a good fashion style to you?’ rated on a 5-point Likert-type scale (1 = *very unimportant*, 5 = *very important*).

##### Tactile hyper-sensitivity

A Clothing Questionnaire was designed to measure tactile sensitivity and behaviours related to clothing. This questionnaire contained 20 items rated on a 5-point Likert-type scale (1 = *never*; 5 = *always*). Twelve items were adapted from the tactile and proprioception domains of the Glasgow Sensory Questionnaire 70-item version (GSQ; Robertson, 2012) and Adult Routines Inventory (Evans et al., 2017). Six additional clothing-related items were developed by the second author (D.B.) and MSc students working on a related project, based on the literature and existing measures. Due to this study focusing on the impacts of hyper-sensitivity, six items (2, 3, 5, 13, 14 and 16) were selected following Principal Component Analysis (see Supplemental Material) to create a tactile hyper-sensitivity subscale (see Appendix 1) which had an acceptable level of reliability (α = 0.77) and corrected item-total correlations > 0.4 (Field, 2017). Participants’ scores for these six items were summed to create the dependent variable ‘tactile hyper-sensitivity’, with total scores ranging from 6 to 30 and a higher score indicating a higher level of hyper-sensitivity.

##### Appearance dissatisfaction

The Centre for Appearance Research Valence Scale (CARVAL; Moss & Rosser, 2012) was used to measure appearance dissatisfaction. This scale includes eight items (e.g. ‘I don’t like the way I look’) rated on a 6-point Likert-type Scale (1 = *strongly disagree*; 6 = s*trongly agree*). Total scores range from 8 to 48, with higher scores suggesting a more negative evaluation of one’s appearance. In this study, it demonstrated an excellent level of reliability (α = 0.95).

##### Appearance awareness

The Centre for Appearance Research Salience Scale (CARSAL; Moss & Rosser, 2012) was used to measure appearance awareness. This scale includes five items (e.g. ‘I am usually conscious of my appearance’) rated on a 6-point Likert-type Scale (1 = *strongly disagree*; 6 = *strongly agree*). Total scores range from 5 to 48, with higher scores suggesting higher levels of conscious awareness of one’s appearance. In this study, the scale demonstrated good reliability (α = 0.88).

##### Self-esteem

The Rosenberg Self-Esteem Scale (RSES; Rosenberg, 1965) was used to measure self-esteem. This scale comprises 10 items (e.g. ‘I feel that I’m a person of worth’) rated on a 4-point Likert-type scale (1 = *strongly disagree*; 4 = *strongly agree*). Total scores range from 10 to 40, with higher scores indicating higher self-esteem. In previous research, it has demonstrated good or excellent internal consistency and good test–retest reliability, including among autistic adults (Nguyen et al., 2020; Zimmerman et al., 2017). In this study, it had an excellent level of reliability (α = 0.93).

#### Qualitative

Participants completed four open-ended questions written by the first author (A.F.K.) to expand on their experiences of living with clothing-related tactile sensitivity and clothing as a form of self-expression: ‘Is finding clothes that fit your sensory needs difficult for you? Please provide examples of this’, ‘Have your sensory issues impacted/limited your fashion style? Please provide examples of this’, ‘Do you feel that the clothes you wear help express who you are as a person?’ and ‘Have clothing-related sensory issues impacted your life and self-esteem? If so, in what way?’.

### Procedure

The survey was completed online via Qualtrics (https://www.qualtrics.com). After reading the information sheet and providing consent, participants completed the demographics questionnaire, Clothing Questionnaire, CARVAL and CARSAL (Moss & Rosser, 2012), RSES (Rosenberg, 1965), CATI (English et al., 2021), the closed question on importance of fashion style and four open-ended questions on clothing-related tactile sensitivity. Participants also completed the Anxiety Scale for Autism-Adults (Rodgers et al., 2020) and WHOQOL-BREF (The WHOQOL Group, 1998) which were not analysed in this study. The median time to complete all questionnaires was 17.68 min. Finally, participants were given the opportunity to provide their email address to participate in the prize draw.

### Analysis

The quantitative data was analysed using IBM SPSS Statistics 28 and the PROCESS macro v4.2 for SPSS (Hayes, 2017). The prevalence of tactile hyper-sensitivity in this sample was calculated by summing the number of participants who responded ‘often’ or ‘always’ for the six items of the tactile hyper-sensitivity scale. Response frequencies for the closed question on the importance of having a good fashion style were also calculated. As the responses to the closed question were considered ordinal data, non-parametric Spearman’s correlations exploring the relationship between importance of fashion style and appearance awareness and dissatisfaction were conducted. Descriptive statistics, bivariate Pearson’s correlations, simple regression analysis and moderation analyses were conducted to test the three study hypotheses. Initial screening of the data determined that it met the assumptions of linearity, homoscedasticity, independence and normality.

Analysis of the open-ended questions was undertaken by the first author (A.F.K.) using Braun and Clarke’s (2006, 2022) methodology for reflexive thematic analysis. An inductive approach was used to capture semantic and latent patterns of meaning across the dataset. The six phases of analysis followed were: data familiarisation by re-reading transcripts; coding data; generating initial themes by clustering codes into broad patterns of meaning; developing and reviewing themes; defining and naming themes; and writing the report. The development and interpretation of codes and themes was discussed with the second author (D.B.) throughout the process.

### Positionality statement

This research project was conducted by non-autistic researchers. The authors’ views align with the neurodiversity paradigm, which conceptualises neurodivergence as a natural and valuable variation of human cognition (Walker, 2014).

### Participatory methods statement

The research questions were developed in line with the research priority of the autistic community to better understand sensory processing differences which can negatively affect autistic people in order to create more sensory-friendly environments (Autistica, 2016), and based on first-person accounts of autistic people found online. An autistic researcher provided feedback on the initial interpretation of the results.

## Results

### Quantitative results

**Figure 1.**
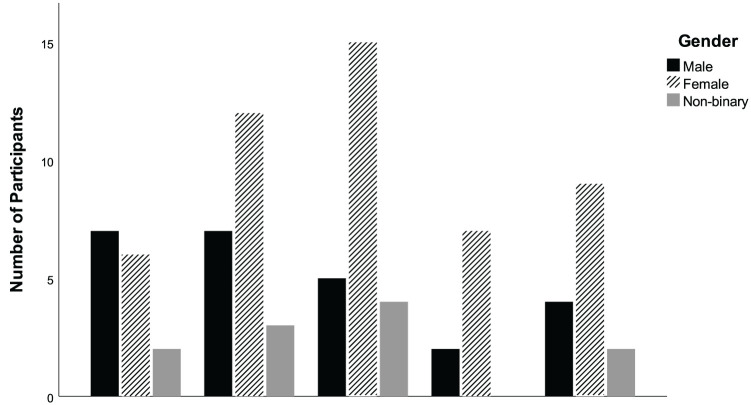
Importance of fashion style broken down by gender.

Participants of all genders reported a range of different opinions towards how important a good fashion style was to them (see Figure 1). Further analyses exploring gender differences were not possible due to low participant numbers when split into subgroups. There was a significant correlation between responses to the importance of fashion style and appearance awareness (*r_s_* = 0.60, *p* < 0.001) confirming that individuals who rated fashion as important to them were more self-aware of their appearance. There was no significant correlation between appearance dissatisfaction and importance of fashion style (*r_s_* = −0.10, *p* = 0.343).

Regarding the frequency of hyper-sensitivity among participants, 73.2% reported they regularly avoid wearing certain types of clothing, 50% dislike having a haircut, 55.8% cut labels off clothes, 46.5% avoid wearing clothing items with seams that will contact their skin, 48.8% have difficulties adapting to new clothing items and 48.8% avoid wearing constricting clothes or shoes.

The zero-order correlation matrix for the key variables and the descriptive statistics are presented in Table 1.

**Table 1.** Descriptive statistics and correlation matrix for predictor and outcome variables.

Variable	M	SD	1	2	3	4
1. Tactile hyper-sensitivity	20.87	5.00	-			
2. Appearance dissatisfaction	30.98	9.42	0.21*	-		
3. Appearance awareness	19.29	5.70	-0.07	-0.11	-	
4. Self-esteem	23.53	6.36	-0.22*	-0.64**	-0.16	-

* *p* < 0.050, **p < 0.001.

A simple linear regression examined the predictive relationship between tactile hyper-sensitivity and appearance dissatisfaction. The analysis indicated that the model was significant (*F*(1, 84) = 3.95, *p* *=* 0.05), with tactile hyper-sensitivity explaining 3.4% (*R*^2^ adjusted = 0.034) of the variance of appearance dissatisfaction. The effect size (*f*^2^ = 0.05) indicated a small effect. Higher tactile hyper-sensitivity was associated with higher appearance dissatisfaction (β = 0.40, *p* = 0.050).

A moderation analysis with centred variables examined the relationship between the predictor variable ‘tactile hyper-sensitivity’, dependent variable ‘self-esteem’ and moderator ‘appearance dissatisfaction’. The moderation model was significant (*F*(3, 81) = 22.52, *p* < 0.001) and explained 45.5% of the variance in self-esteem (*R*^2^ = 0.455). The interaction was significant (*b* = −0.026, 95% CI = [−0.048, −0.003], *t* = −2.28, *p* = 0.025), indicating that there is a predictive relationship between hyper-sensitivity and self-esteem moderated by appearance dissatisfaction, with a large effect size (*f*^2^ = 0.83). When appearance dissatisfaction is low (*b* = 0.074, 95% CI = [−0.191, 0.338], *t* = 0.55, *p* = 0.581) or at the mean value (*b* = −0.169, 95% CI = [−0.387, 0.049], *t* = −1.55, *p* = 0.126), there is a non-significant relationship between hyper-sensitivity and self-esteem. However, when appearance dissatisfaction is high, there is a significant negative relationship between hyper-sensitivity and self-esteem (*b* = −0.412, 95% CI = [−0.752, −0.073], *t* = −2.42, *p* = .018), indicating that the negative predictive relationship between hyper-sensitivity and self-esteem only emerges in people with higher levels of appearance dissatisfaction, not those with low or mean levels.

Another moderation analysis examined the relationship between the predictor ‘appearance dissatisfaction’, dependent variable ‘self-esteem’ and moderator ‘appearance awareness’. The moderation model was significant (*F*(3, 81) = 24.79, *p* < 0.001), explaining 47% of the variance. However, the interaction was non-significant (*p* = 0.134) indicating that appearance awareness does not influence the relationship between appearance dissatisfaction and self-esteem. Due to this, another model without the interaction was conducted. This hierarchical regression was found to be significant (*F*(2, 82) = 35.48, *p* < 0.001) and accounted for 45.1% (*R*^2^ adjusted = 0.451) of the variance in self-esteem, with a large effect size (*f*^2^ = 0.86). Higher appearance dissatisfaction (β = −0.67, *p* < 0.001) and higher appearance awareness (β = −0.23, *p* = 0.006) were significant predictors of lower self-esteem.

### Qualitative results

Three main themes, with five sub-themes, relating to the impacts of clothing-related tactile sensitivity on autistic adults’ lives were generated. ‘Negative consequences’ captures the direct negative impacts of clothing-related tactile sensitivity. ‘Managing clothing-related tactile sensitivity’ captures how autistic adults cope with and adapt to living with clothing-related tactile sensitivity. ‘The emotional value of clothing’ captures how clothing has an important emotional role for autistic adults and positively impacts aspects of their well-being despite the various negative impacts of hyper-sensitivity.

#### Theme 1: Negative consequences

Participants described negative experiences of living with clothing-related tactile sensitivity and how this directly affects key aspects of their lives.

##### Physical discomfort

Sensitivity to certain clothing items or characteristics caused clothing to be a potential source of physical discomfort for autistic adults. Touching or wearing aversive clothes could cause participants to experience itchiness, over-heating, scratchy or tickly sensations, and feelings of being constricted:

. . . trying on clothes is the worst (because it feels like bugs are all over my skin and I want to get it off as soon as possible). (P1)

I struggle to find tops that don’t press on my neck which makes me feel like I’m choking. (P2)

As a result, participants described certain clothing items as physically unpleasant and difficult to wear. Commonly disliked characteristics included labels, seams, and itchy or synthetic fabrics such as wool and polyester. Some participants also disliked tight clothing such as jeans and tops with long sleeves or tight necklines. Others reported difficulties with socks, shoes, underwear, shimmery or sparkly fibres, and decorative elements like sequins:

A lot of fabrics feel awful, particularly synthetic or scratchy things. I can’t deal with long sleeves at all. (P3)

Seams and labels are the main issues. (P4)

I cannot wear clothes of certain fabrics because the feel is horrible, like velvet and fake/cheep [*sic*] corduroy. (P5)

##### Decreased emotional well-being

Wearing aversive clothing was also described by participants as emotionally distressing, as this triggered feelings of irritability, overwhelm, stress, distress and anxiety:

I really can’t wear floaty/loose fitting clothing cause [*sic*] I find it so tickly and it makes me really irritable. (P6)

I cannot wear anything with sparkly fibres in, or anything fluffy because it upsets me, as they feel too much on my skin. (P7)

Being unable to wear the clothes they would like to due to their clothing-related tactile sensitivity also resulted in some participants feeling less confident, and unhappy with their appearance:

There is nothing fashionable that fits my needs – I feel like I dress like an old man. (P8)

I like the look of summer dresses and fancy frocks but I just can’t wear them cos [*sic*] they feel too odd and floaty on my body . . . it all limits my fashion I end up looking like a tomboy even though I’m not . . . (P9)

These accounts reflected a sense of ‘missing out’ on the opportunity to enjoy clothing and use this as a form of self-expression, which often led to feelings of frustration and dissatisfaction:

It can be frustrating not being able to just wear whatever I want, especially seeing other people being able to do so and express themselves through their clothing. (P10)

I don’t feel that my clothes express who I really am, but I feel trapped in my ‘safe clothes’ and would feel silly trying anything else now. (P11)

Being non-binary, I would love to explore fashion more and help my appearance match how I am feeling in my gender but I can’t because the clothes I want to wear make me have a meltdown. It is so unfair. (P12)

##### Barrier to daily life

The discomfort caused by clothing-related tactile sensitivity also impacted autistic adults in their day-to-day life. Participants reported this as a barrier to engaging with work, education and social events. Wearing aversive clothing during these activities could have negative practical consequences such as difficulties concentrating, as well as negative emotional consequences such as feeling self-conscious:

Sensory issues definitely impacted my life, they can be terribly distracting so when I had to wear business casual and waistbands for old office jobs it would have upset my performance. (P13)

Thinking back to school uniform, I also struggled to wear it and felt very much like I couldn’t be myself while dealing with stiff fabrics, labels, etc. (P14)

When it came to cultural/religious events and I was expected to wear certain items of clothing, like sheer fabrics with a lot of embroidery and stitching, I would feel a lot of discomfort and subsequently struggle to socialise and enjoy myself. At the time, I thought I was just being dramatic and unreasonable, which impacted my self-esteem. (P15)

. . . sometimes at formal events if my clothes are very uncomfortable because comfortable fancy clothes are difficult to find then I am extra anxious and self conscious. (P16)

#### Theme 2: Managing clothing-related tactile sensitivity

The persistence of tactile sensitivity throughout life meant that participants had to find ways of managing their tactile sensitivity towards clothing, including restricting clothing purchases to items they knew would not cause difficulties and finding compromise between their sensory needs and preferred clothing.

##### Coping strategies

In order to prevent or cope with clothing-related tactile sensitivity, participants had developed various strategies. This included altering clothing, going to shops to examine clothing items before buying them to ensure they were sensory-friendly and buying multiple of the same sensory-friendly item or similar items from the same brand:

I need to see and feel something before buying it. (P17)

If I find something comfortable I’ll buy it in several colours/patterns. (P3)

I cut the labels out of almost all my tops. (P4)

In addition, participants reported avoiding buying and wearing clothing items which they were aware would cause physical discomfort:

I mostly stick to cottons and avoid synthetic materials. (P15)

If I don’t like the feel or look of the material I won’t buy it. (P18)

Positively, this suggests that autistic adults develop coping strategies which enable them to successfully manage their clothing-related sensitivity. Simultaneously, participants’ responses implied that adapting one’s life around preventing and coping with this can be time-consuming and costly. For instance, purchasing clothing of higher quality to avoid seams or synthetic fabrics is more expensive, and examining clothing in person involves spending additional time searching for specific items and travelling to certain shops:

I like to buy the same items I bought before but often can’t find them. (P19)

I also dislike seams so I sometimes have to pay more for seamless clothes like socks. (P5)

Furthermore, avoiding certain types of clothing limited participants’ options. Autistic adults described difficulties finding clothing in bigger sizes and alternative fashion styles in particular:

I can only wear leggings, t-shirts and sleeveless fleeces. (P8)

Yes, especially as a midsize person into alternative fashion, I struggle to find things my size; almost everything is suffocating and made of elastane, making me itchy. (P20)

##### Compromise

An internal evaluation around deciding whether to prioritise comfort or fashion style was reflected within participants’ accounts:

If I’m having a bad day, wearing something that overstimulates me makes it worse, but putting on something more accommodating to my sensory issues makes me feel bad about my body. (P7)

Wanting to look attractive . . .; to ‘fit in’; to be safe; to present well in professional or near-professional situations, to act ethically, to appear approachable, dress quickly, to shop for clothes quickly . . . Given all these, I’ll accept a level of discomfort if my clothes ‘tick’ other boxes. (P21)

For numerous participants, comfort was a priority and necessity when choosing clothing in order to avoid the negative consequences of clothing-related tactile sensitivity:

I go for comfort over fashion. (P22)

Every item of clothing is chosen for how it feels! (P23)

Consequently, if participants were unable to find clothing in their desired style which met their sensory needs, they had to compromise by sacrificing their desired appearance for comfort:

I wear what is comfortable rather than what I’d like to wear for appearance. (P24)

I wear my style as long as material feels nice and suit me. (P25)

Altogether, participants’ reflections suggest that the impact of clothing-related tactile sensitivity extends beyond the direct negative consequences and includes the need to adapt one’s life in order to avoid possible adverse effects.

#### Theme 3: The emotional value of clothing

Throughout the dataset, it was apparent that clothing was not only perceived as a necessary physical object or source of discomfort, but also as having an important emotional role and value. Fashion and clothing were considered by several participants to be a way of expressing parts of their identity such as their interests, personality and gender identity:

I try my hardest to buy clothes that fit my aesthetic and help me show who I am to the world as best as possible. (P26)

The clothes I wear often help me express what I’m feeling (emotionally, in terms of gender presentation, etc.). (P15)

I still wear band t shirts and similar items that reflect my tastes, etc. (P27)

Expressing oneself through clothing had a positive impact on these participants’ self-esteem and emotional well-being as it led to them feeling more confident, happier and more satisfied with their appearance:

I often like wearing my favourite clothes (e.g. I get obsessed over a particularly [*sic*] jacket and wear it for weeks) because it expresses who I am and makes me feel fluffy inside. (P1)

I collect T-shirts from events I’ve attended and enjoy people asking me about the stories behind each one. (P28)

Lots of loud, bright colours express and help me to feel more positive about myself. (P14)

I own 4 dresses with cats on them as that makes me feel happy and safe. I guess the bright, bold things I wear give off weird but friendly and enthusiastic vibes, which is very much me. (P29)

Therefore, despite the negative impacts of clothing-related tactile sensitivity on various aspects of autistic adults’ lives, clothing itself can have a notable positive impact.

## Discussion

The present study provides novel insight into autistic adults’ experiences of living with clothing-related tactile sensitivity. First, responses to the Clothing Questionnaire suggest that tactile sensitivity towards clothing is common among autistic adults, with estimates ranging from 48.8% to 73.2% across hyper-sensitivity items. This provides support for the persistence of tactile sensory sensitivity into adulthood (e.g. MacLennan, O’Brien et al, 2022). Generally, autistic adults reported disliking labels, seams, itchy and synthetic fabrics and tight clothing items. This aligns with the preferences described in previous qualitative research (Kyriacou et al., 2021; MacLennan, O’Brien, et al., 2022). As clothing is a key part of a person’s daily sensory environment, this highlights the importance of understanding clothing-related tactile sensitivity among autistic people.

The regression analysis indicated that higher levels of tactile hyper-sensitivity predict higher appearance dissatisfaction among autistic adults. The moderation analysis then revealed that higher levels of tactile hyper-sensitivity also predict lower self-esteem in autistic adults who are highly dissatisfied with their appearance. This suggests that tactile hyper-sensitivity is a factor contributing to negative self-perception. In this study, autistic adults had a more negative evaluation of their appearance (see Table 1) compared to the general population (e.g. Females 19.7 (7.6); Moss & Rosser, 2012) and adults with visible appearance differences (e.g. Females 22.5 (7.8); Moss et al., 2014) in previous studies. This has important implications for understanding low self-esteem among autistic people (e.g. Nguyen et al., 2020). The qualitative findings of this study complement these quantitative findings. The first theme ‘Negative consequences’ highlights how clothing-related tactile sensitivity directly affects autistic adults’ physical comfort, emotional well-being and social engagement. Within this, some participants reported that their self-esteem and appearance satisfaction were negatively affected by being unable to wear the clothes they would like to. Due to the sample size being too small for mediation analysis (Fritz & MacKinnon, 2007), it remains quantitatively unclear if the relationship between clothing-related tactile sensitivity and self-esteem is a direct result of appearance dissatisfaction, or solely moderated by this. Ultimately, these findings suggest it is important for mental health professionals working with autistic adults to consider and explore how tactile hyper-sensitivity may be interfering with their appearance satisfaction and self-esteem.

Moss and Rosser (2012) suggested that appearance awareness is unrelated to the emotional impact of the information brought to consciousness and instead it may be used to guide behaviours related to appearance without leading to distress. However, when appearance dissatisfaction is high and constant, individuals with heightened awareness may be more at risk of experiencing appearance concerns and mental distress (Moss et al., 2014; Moss and Rosser, 2012). The zero-order correlations indicated that appearance awareness was not associated with any of the other main variables nor did it moderate the relationship between appearance dissatisfaction and self-esteem in this study. However, the final hierarchical regression model showed that appearance awareness predicts self-esteem when appearance dissatisfaction is accounted for. In this study, autistic adults who considered having a good fashion style important were more aware of their appearance. Future quantitative research could investigate whether other functions of clothing – such as expressing individuality, comfort and concealing one’s body (Tiggemann & Lacey, 2009) – or conforming to socio-cultural expectations is linked to greater appearance awareness in autistic adults. This is especially pertinent to individuals who mask as they are more likely to conceal aspects of their identity, including potentially their appearance, which may lead to mental health difficulties and a decreased sense of authenticity (Evans et al., 2023; Hull et al., 2017).

In addition to the emotional impacts, clothing-related tactile sensitivity was described as a barrier to engagement with activities such as employment and formal social events. It is important to note that only 29% of autistic adults in the United Kingdom are in employment (Office for National Statistics, 2022). Barriers to gaining and maintaining employment for autistic adults across sectors include stigma, executive function difficulties and socio-communication difficulties such as understanding social norms (Harmuth et al., 2018). Previously reported sensory barriers in the workplace include bright lights, constant background noise and crowded environments (Jones, 2023; Wood & Happé, 2023). The present study indicates that clothing can also be a workplace barrier. Adjustments to dress codes and uniforms in the workplace and education settings can be a reasonable adjustment under the Equality Act 2010 (Equality Act, 2010). Nonetheless, Ambitious about Autism’s recent ‘More Than Just Clothes’ campaign highlighted that 1 in 3 autistic people decide not to apply for a job because of a mandatory uniform policy (Ambitious about Autism, 2024). Rather than being considered as an afterthought, clothing-related tactile sensitivity should be considered when creating uniform policies to anticipate this potential barrier to employment, and improve autistic employees’ well-being and ability to succeed at work.

Autistic adults in this study managed their clothing-related tactile sensitivity through various coping strategies such as examining items in stores, buying multiple of the same sensory-friendly item and avoiding items of clothing which cause discomfort. Kyriacou et al. (2021) also identified examining clothing in shops and avoiding certain textiles as coping strategies for dealing with uncomfortable fabrics. Living with clothing-related tactile sensitivity meant that participants often had to choose between prioritising comfort and fashion style when choosing what to wear. This suggests clothing-related tactile sensitivity has other, more inadvertent impacts on autistic adults’ lives through the choices and adaptations they have to make to manage this – in addition to the more direct negative physical and emotional consequences of touching disliked clothing stimuli. Moreover, these strategies may result in hyper-sensitivity towards clothing being overlooked, particularly among undiagnosed autistic adults. Several participants’ accounts imply that it is not that they are unaffected by clothing-related tactile sensitivity as such, instead they are able to prevent this negative sensory experience from actively impacting them by avoiding exposure to aversive clothing.

In line with other population groups (Hester & Hehman, 2023; Tiggemann & Lacey, 2009), clothing was described an important form of self-expression for autistic adults. In particular, wearing clothing that expresses aspects of one’s identity, such as interests, personality and gender, was important for feeling confident and being happy with one’s appearance. In contrast, being unable to wear the clothing one would like due to clothing-related tactile sensitivity was associated with feelings of frustration, ‘missing out’ and dissatisfaction with one’s appearance; and appearance dissatisfaction accordingly predicted lower self-esteem among participants. Future qualitative research could explore how autistic adults of different genders explore or affirm their gender identity through clothing.

Finally, many of the aforementioned coping strategies used by autistic adults ultimately aimed to increase the amount of clothing options available for them. Retail shops and shopping centres are often inaccessible for autistic adults due to the presence of intense, multi-sensory input (MacLennan, Woolley, et al., 2022) and the fashion industry primarily produces ready-to-wear garments which do not cater to a wide range of needs, including those of physically disabled people (Esmail et al., 2022) or autistic people with hyper-sensitivity. As a result, autistic people face significant barriers to finding and purchasing clothing that meets their sensory needs. Positively, the number of adaptive clothing retailers and adaptive clothing lines in mainstream brands is gradually increasing (Esmail et al., 2020). Collaboration between clothing brands, the autistic community and researchers is fundamental to help designers and manufacturers create evidence-based sensory-friendly clothing to maximise benefits such as increased self-expression and reduce negative effects of clothing-related tactile sensitivity.

### Strengths and limitations

The present study builds on previous research identifying clothing which autistic adults find comfortable and uncomfortable by exploring their perception of clothing and the impacts of clothing-related sensitivity. Research on the impacts of clothing-related hyper-sensitivity on well-being and daily functioning is extremely limited and mostly qualitative. A mixed-methods design was used in this study to ensure autistic adults’ experiences were captured as accurately as possible while also examining these constructs quantitatively.

The sample was diverse in terms of gender, age, employment, co-occurring conditions and opinions regarding the importance of fashion. However, autistic people of colour were under-represented. Autistic adults without a formal diagnosis were excluded due to concerns around the reliability and accuracy of findings, as well as those with an intellectual disability. There was no community involvement during the design or implementation of this study due to time constraints and lack of funding – which future research should address and include. Notably, the study captured the views and experiences of 86 autistic adults through the analysis of open-text responses, allowing for a variety of experiences of clothing-related tactile sensitivity within the autistic community to be reflected and mitigating the risk of bias. Although clothing-related tactile sensitivity was common among participants, not all of them were impacted equally by this. While some participants – such as those dissatisfied with their appearance – were significantly affected by this emotionally and in their day-to-day life, others were less affected. Moreover, participants preferred and disliked very different clothing items. For example, some participants preferred loose clothing as they found tight clothing ‘suffocating’, whereas other participants found loose clothing uncomfortable as this ‘moves about too much’ and can be ‘tickly’. Furthermore, tactile sensory stimuli can also be enjoyable and comforting for autistic adults when this has a physical feel they like (MacLennan, O’Brien, et al., 2022; Robertson & Simmons, 2015). This highlights the individualistic nature of sensory sensitivity and, consequently, the importance of autistic people and those supporting them understanding their unique sensory profile.

Due to time constraints and a lack of measures examining clothing-related hyper-sensitivity, items were adapted from the GSQ 70-item version (Robertson, 2012) to examine this construct in this study. Although this had acceptable reliability and items aligned with the experiences of participants in this and previous studies (e.g. MacLennan, O’Brien, et al., 2022), a more rigorous measure needs to be created to ensure findings in this research area are accurate and generalisable. In particular, great care must be taken when formulating questions about participants’ experiences of highly subjective concepts such as ‘a good fashion style’, as a participant may interpret this in several ways including fitting socio-cultural standards of appearance or meeting their desired personal style.

## Conclusion

This study demonstrates that clothing-related tactile sensitivity has significant, widespread effects on autistic adults’ lives including their appearance satisfaction, self-esteem, and their clothing options and choices. In turn, this study highlights the importance of access to sensory-friendly clothing and self-expression through clothing for autistic adults’ comfort, engagement with daily life and self-confidence. These findings have important implications for mental health professionals, clothing brands and ensuring equity in the workplace.

## Supplemental Material

sj-pdf-1-aut-10.1177_13623613251366882 – Supplemental material for ‘I feel trapped in my safe clothes’: The impact of tactile hyper-sensitivity on autistic adults Supplemental material, sj-pdf-1-aut-10.1177_13623613251366882 for ‘I feel trapped in my safe clothes’: The impact of tactile hyper-sensitivity on autistic adults by Amanda Ferrer Knight and Deirdre Birtles in Autism

## Appendix 1

### Clothing questionnaire (hyper-sensitivity items)

2. Do you avoid wearing certain types of clothes (e.g. ones made from scratchy material like wool)?
3. Do you dislike having a haircut (e.g. because little bits of hair go down your back)?
5. Do you cut the labels out of your clothes?
13. Do you avoid wearing items of clothing with seams that will contact your skin (e.g. in socks or underwear)?
14. Do you have difficulty adapting to new items of clothing?
16. Do you avoid wearing clothes or shoes that are constricting (e.g. tight shirt collars, ties, belts, waistbands)?
