# Supplementary material for: ‘I feel trapped in my safe clothes’: The impact of tactile hyper-sensitivity on autistic adults
Source: Autism. 2025 Oct 7;29(11):2727–40. doi: 10.1177/13623613251366882 (PMC12531387; doi:10.1177/13623613251366882)

Supplemental Material: Information About Factor Analysis of the Clothing Questionnaire

A Principal Components Analysis on the 20 items of the Clothing Questionnaire was performed to determine whether a factor assessing tactile hyper-sensitivity could be identified (N = 86). The Kaiser-Meyer-Olkin measure of sampling adequacy (0.62), Bartlett test for sphericity ($p < .001$) and determinant statistic (.008) indicated that the data was suitable for factor analysis. Following a varimax rotation an eight-component solution was identified, explaining 67.9% of the total variance. Six items (2, 3, 5, 13, 14 and 16) loaded together into one factor explaining 18.1% of the total variance. Items 2, 3 and 5 were adapted from the Glasgow Sensory Questionnaire 70-item version (Robertson, 2012). Items 13, 14 and 16 were created as part of this study. These six items assessed hyper-sensitivity in the tactile domain and, consequently, were selected to create a 'tactile hyper-sensitivity' subscale for the current study.

Supplemental Material: Frequency Analysis

Table S1

Frequency Analysis of the Clothing Questionnaire

Item	Frequency				
	Never	Rarely	Sometimes	Often	Always
1. Do you dislike the physical sensation you get when people hug you?	5.8%	22.1%	38.4%	26.7%	7.0%
2. Do you avoid wearing certain types of clothes (for example, ones made from scratchy material like wool)?	1.2%	5.8%	19.8%	30.2%	43.0%
3. Do you dislike having a haircut (for example, because little bits of hair go down your back)?	14.0%	10.5%	25.6%	26.7%	23.3%
4. Do you feel pain very easily?	5.8%	26.7%	32.6%	23.3%	11.6%
5. Do you cut the labels out of your clothes?	10.5%	12.8%	20.9%	30.2%	25.6%
6. Do you like the feeling of vibrations against your skin?	17.4%	27.9%	31.4%	19.8%	3.5%
7. Do you like the feeling when someone presses forcefully into your muscles/back? This could be likened to deep pressure massage. [a]	14.0%	10.5%	31.4%	16.3%	26.7%
8. Do you find it difficult to tie your shoelaces or button up your clothes?	31.4%	22.1%	23.3%	17.4%	5.8%
9. Do you find that you are able to go outside without a coat or jacket when other people think that it is too cold?	16.3%	22.1%	26.7%	27.9%	7.0%
10. Does your body ever feel 'numb' – like you can't feel anything against your skin?	27.9%	31.4%	26.7%	14.0%	0.0%
11. Are you concerned with dirt, cleanliness or neatness?	1.2%	10.5%	33.7%	33.7%	20.9%
12. Do you notice imperfections in objects, like spots/stains or frays on clothing?	1.2%	5.8%	20.9%	36.0%	36.0%

13. Do you avoid wearing items of clothing with seams that will contact your skin (for example, in socks or underwear)?	10.5%	18.6%	24.4%	30.2%	16.3%
14. Do you have difficulty adapting to new items of clothing?	8.1%	15.1%	27.9%	31.4%	17.4%
15. Do you like to wear tight clothing (for example, close fitting tops, sportswear)?	23.3%	26.7%	30.2%	15.1%	4.7%
16. Do you avoid wearing clothes or shoes that are constricting (for example, tight shirt collars, ties, belts, waistbands)?	11.6%	9.3%	30.2%	26.7%	22.1%
17. Do you like to wear layers of clothing (for example, heavy winter clothes)?	5.8%	25.6%	26.7%	33.7%	8.1%
18. Do you lay your clothes out as part of your dressing routine?	19.8%	19.8%	24.4%	20.9%	15.1%
19. Do you like to wear shirts or tops with short sleeves?	3.5%	12.8%	33.7%	33.7%	16.3%
20. Do you like to put on your clothes in a particular order?	10.5%	15.1%	10.5%	31.4%	32.6%

Note. Items 1 to 10 were adapted from the Glasgow Sensory Questionnaire 70-item version (Robertson, 2012). Items 11 and 12 were adapted from the Adult Routines Inventory (Evans et al., 2017).

[a] One value missing (n = 85).

Supplemental Material: Graphs From Regression and Moderation Analyses

Figure S1

Scatterplot Showing the Relationship Between Tactile Hyper-Sensitivity and Appearance Dissatisfaction

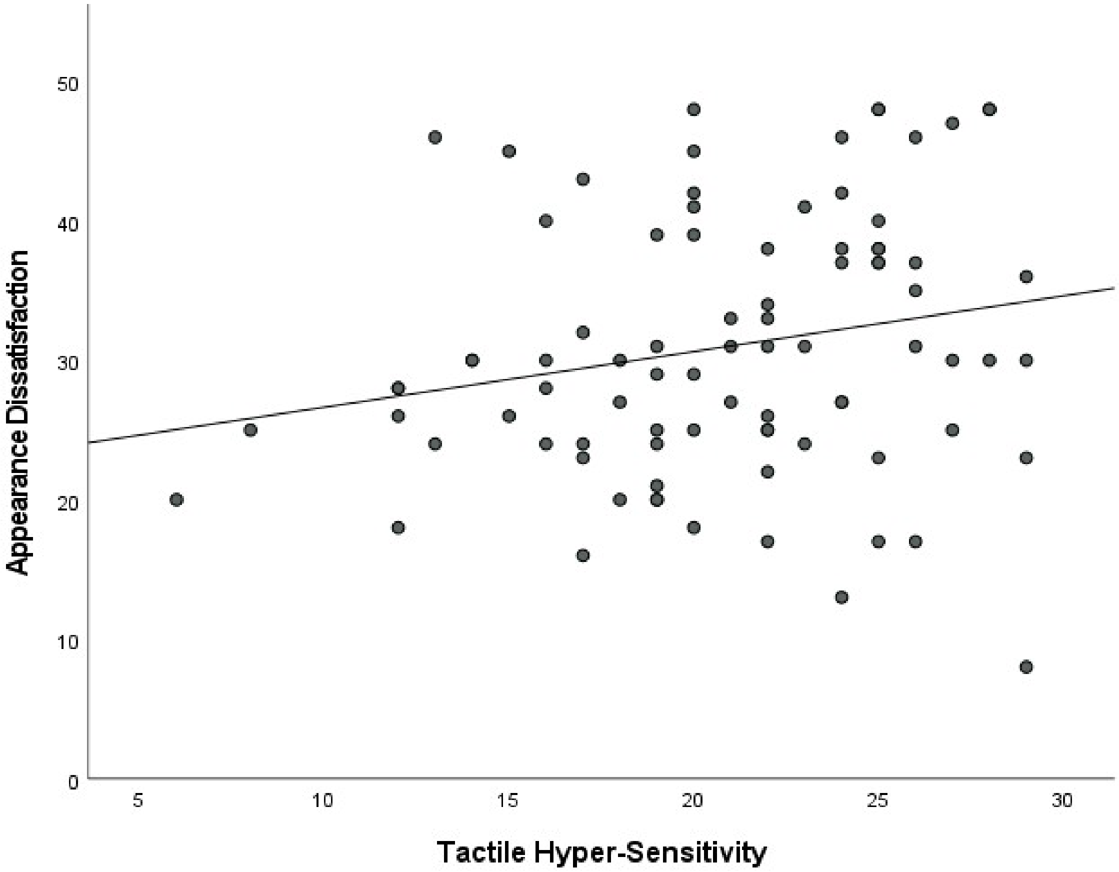

Figure S2

Scatterplot Showing the Relationship Between Appearance Dissatisfaction and Self-Esteem

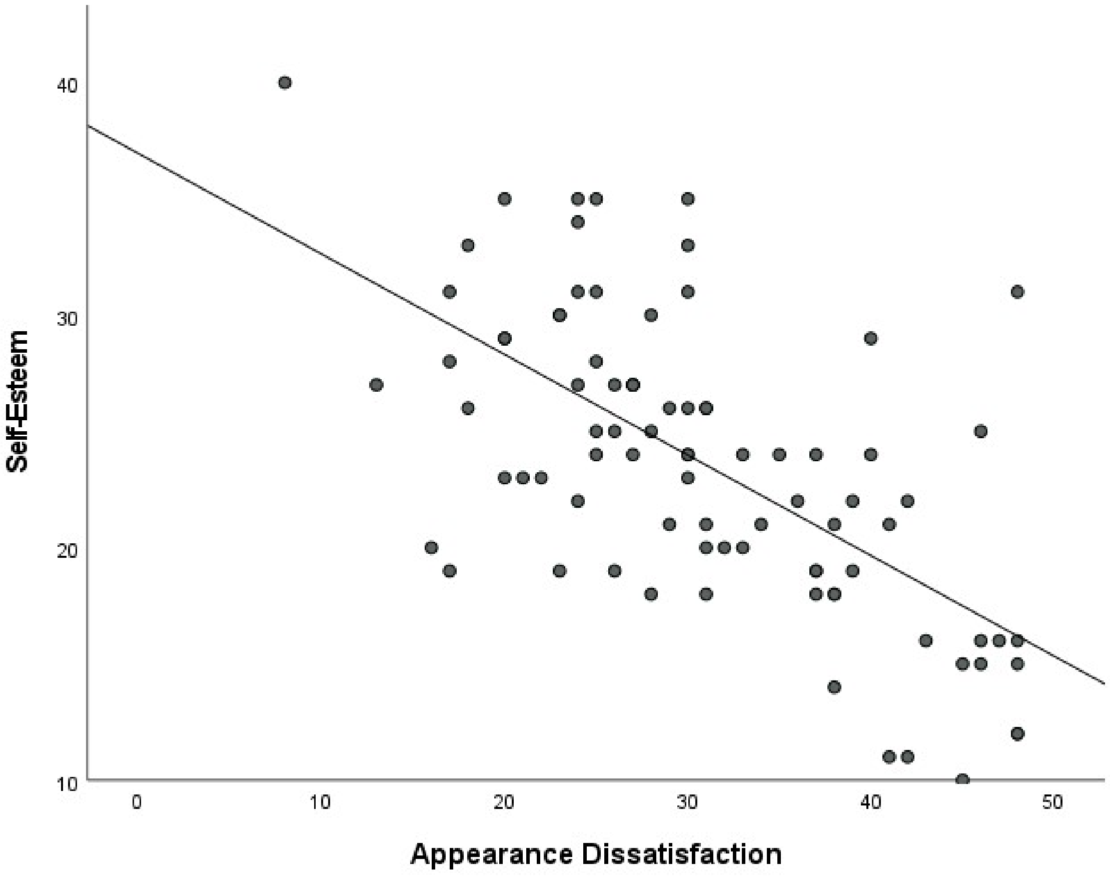

Figure S3

Scatterplot Showing the Relationship Between Appearance Awareness and Self-Esteem

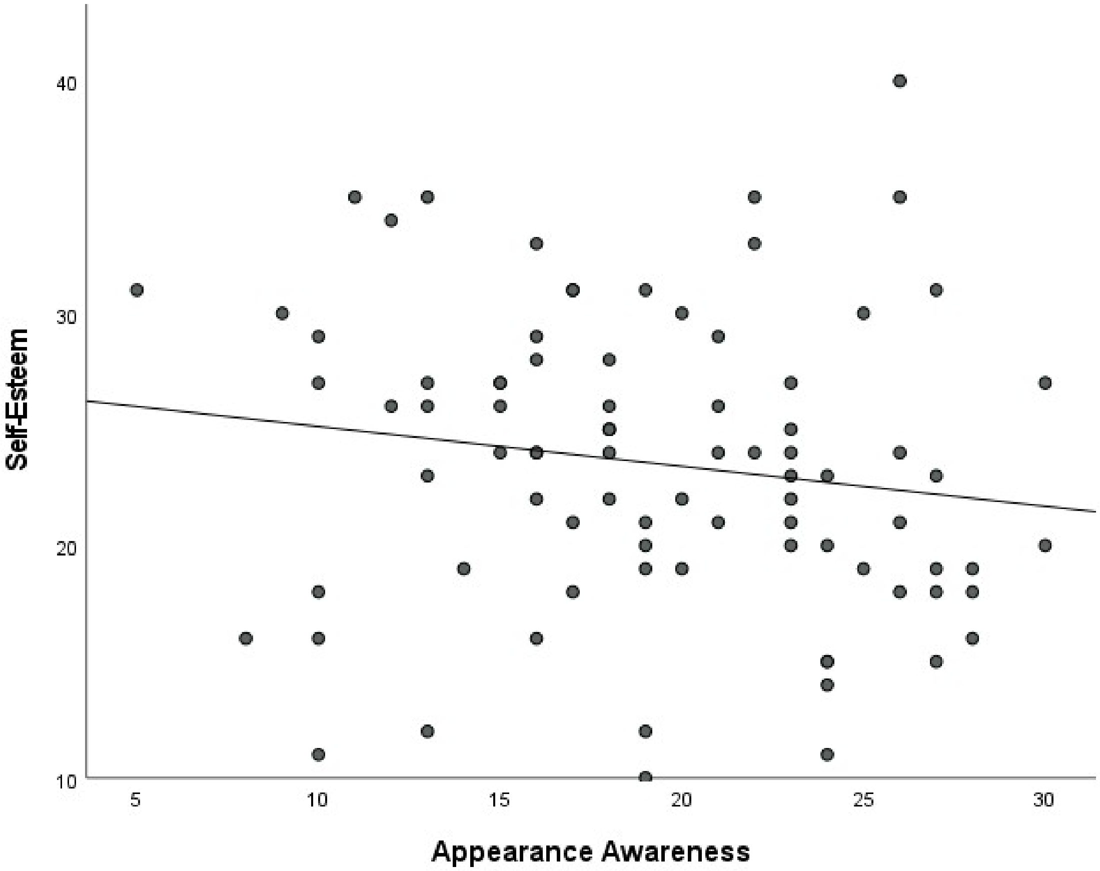

Figure S4

Simple Slopes Plot Showing the Relationship Between Tactile Hyper-Sensitivity and Self-Esteem at Different Levels of Appearance Dissatisfaction

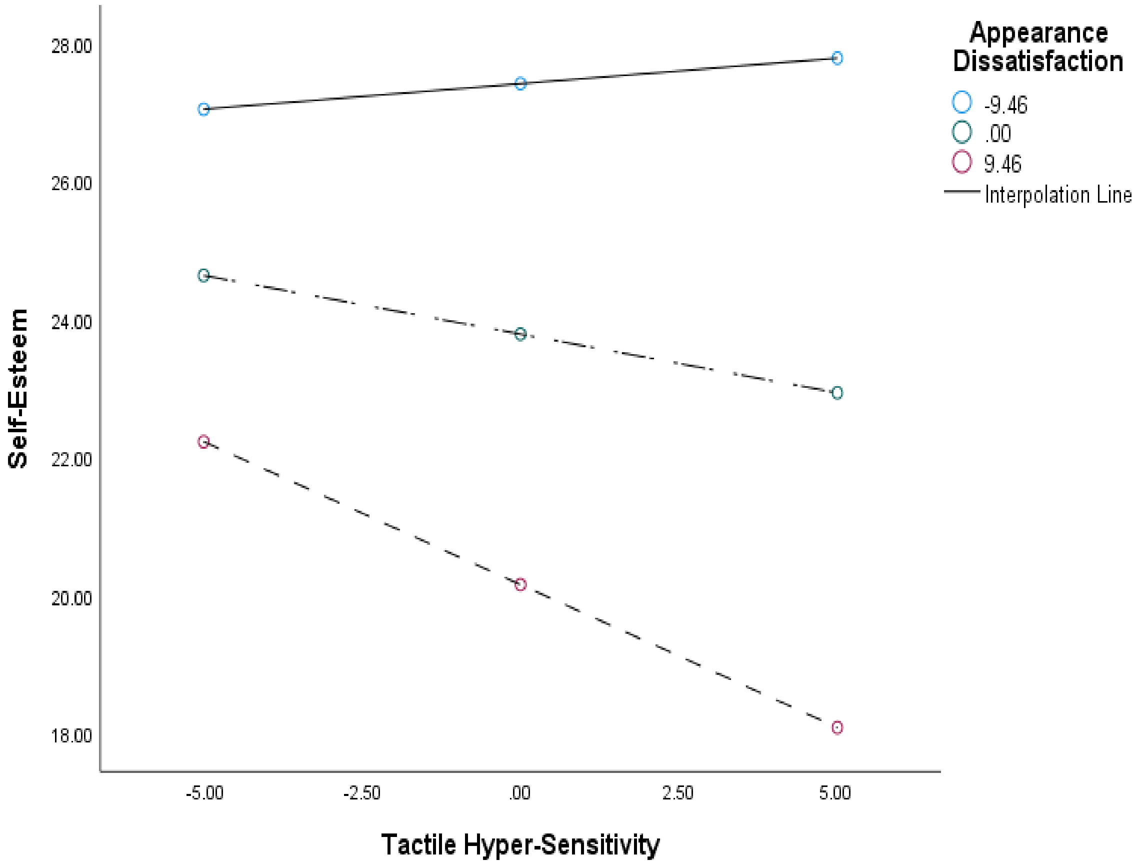

Supplement: sj-pdf-1-aut-10.1177_13623613251366882 – Supplemental material for ‘I feel trapped in my safe clothes’: The impact of tactile hyper-sensitivity on autistic adults [file sj-pdf-1-aut-10.1177_13623613251366882.pdf]
